# Enhancing Precision in HIV Treatment: Validation of a Robust Next-Generation Sequencing System for Drug Resistance Mutation Analysis

**DOI:** 10.3390/diagnostics14161766

**Published:** 2024-08-14

**Authors:** Ashutosh Vashisht, Ashis K. Mondal, Vishakha Vashisht, Sudha Ananth, Ahmet Alptekin, Kimya Jones, Jaspreet K. Farmaha, Ravindra Kolhe

**Affiliations:** 1 Georgia Esoteric and Molecular Biology Laboratory, Department of Pathology, Augusta University, Augusta, GA 30912, USA; avashisht@augusta.edu (A.V.); amondal@augusta.edu (A.K.M.); vvashisht@augusta.edu (V.V.); sananth@illumina.com (S.A.); aalptekin@augusta.edu (A.A.); kjones1@augusta.edu (K.J.); jfarmaha@augusta.edu (J.K.F.); 2 Reagent Sciences Department, Research and Development, Illumina, San Diego, CA 92122, USA

**Keywords:** HIV, drug resistance, next-generation sequencing, antiretroviral therapy, clinical validation

## Abstract

Background: Multidrug-resistant HIV strains challenge treatment efficacy and increase mortality rates. Next-generation sequencing (NGS) technology swiftly detects variants, facilitating personalized antiretroviral therapy. Aim: This study aimed to validate the Vela Diagnostics NGS platform for HIV drug resistance mutation analysis, rigorously assessed with clinical samples and CAP proficiency testing controls previously analyzed by Sanger sequencing. Method: The experimental approach involved the following: RNA extraction from clinical specimens, reverse transcription polymerase chain reaction (RT-PCR) utilizing the Sentosa SX 101 platform, library preparation with the Sentosa SQ HIV Genotyping Assay, template preparation, sequencing using the Sentosa SQ301 instrument, and subsequent data analysis employing the Sentosa SQ Suite and SQ Reporter software. Drug resistance profiles were interpreted using the Stanford HIV Drug Resistance Database (HIVdb) with the HXB2 reference sequence. Results: The Vela NGS system successfully identified a comprehensive array of drug resistance mutations across the tested samples: 28 nucleoside reverse transcriptase inhibitors (NRTI), 25 non-nucleoside reverse transcriptase inhibitors (NNRTI), 25 protease inhibitors (PI), and 10 integrase gene-specific variants. Dilution experiments further validated the system’s sensitivity, detecting drug resistance mutations even at viral loads lower than the recommended threshold (1000 copies/mL) set by Vela Diagnostics. Scope: This study underscores the validation and clinical applicability of the Vela NGS system, and its implementation may offer clinicians enhanced precision in therapeutic decision-making for individuals living with HIV.

## 1. Introduction

The advent of combined antiretroviral therapy (cART) revolutionized human immunodeficiency virus (HIV) management by effectively suppressing viral replication, restoring immune function, and enhancing patients’ quality of life. Despite this advancement, the Joint United Nations Program on HIV and acquired immunodeficiency syndrome (AIDS) and the World Health Organization (WHO) present a very challenging global HIV landscape: in 2022, 39 million individuals were living with HIV infections, 1.3 million newly acquired the virus, and an estimated 630 thousand were facing mortality due to HIV-related issues [1,2]. The juxtaposition of these staggering statistics against the backdrop of therapeutic innovation underscores both the progress made in HIV management and the persistent challenges that demand our attention [3].

Presently, HIV treatment encompasses a diverse arsenal of antiretroviral medications spanning five distinct classes. These medications target various stages of the HIV life cycle: entry inhibitors, such as maraviroc and enfuvirtide, disrupt viral attachment and fusion; nucleoside and non-nucleoside reverse transcriptase inhibitors (NRTIs and NNRTIs) impede viral RNA conversion to cDNA; integrase strand transfer inhibitors (INSTIs) prevent viral DNA integration into the host genome; and protease inhibitors (PIs) hinder viral protein cleavage, thus inhibiting virion maturation [4].

However, despite these therapeutic advancements, the emergence of drug-resistant and multidrug-resistant HIV strains remains a formidable challenge. As highlighted in the latest WHO HIV Drug Resistance Report, an exponential rise in acquired and transmitted drug resistance among antiretroviral treatment-naive individuals has been observed, resulting in cART failure, disease progression, and increased mortality [5]. The emergence of such resistant strains poses a significant barrier to achieving the goal of ending the HIV-1 epidemic as a public health threat by 2030 [6], hence the urgency highlighted by the WHO to combat emerging HIV drug resistance, advocating for novel interventions to counter this emerging threat to global public health. This includes implementing routine viral load monitoring, enhancing treatment adherence, conducting drug resistance genotyping, timely regimen adjustments, and selecting optimal antiretroviral combinations to ensure sustained treatment efficacy and favorable outcomes in the long term.

Next-generation sequencing (NGS) has emerged as a pivotal tool for clinical, research, and public health applications, supplanting Sanger sequencing (SS) as the primary method for gene identification and characterization across diverse organisms and viruses, including HIV [7]. While the SS can detect drug resistance mutations (DRMs) in 20–30% of the viral population, NGS technologies offer superior sensitivity and reproducibility, identifying low-frequency variants down to 5% of the total virus population [8]. NGS HIV drug resistance (HIVDR) assays offer flexibility, allowing the simultaneous sequencing of multiple HIV genes through the use of longer PCR amplicons or the combination of different amplicons before fragmentation and tagmentation during library preparation, facilitating the high-resolution analysis of HIVDR variants [9]. The affordability and accessibility of NGS technology, facilitated by commercial kits and analysis pipelines, have enabled high-throughput, massively parallel sequencing to identify low-abundance variants in various applications, including HIVDR testing [7,10]. However, the majority of NGS platforms available, such as Illumina and Roche, are certified for research use only and, thus, not yet suitable for clinical diagnostic applications. In 2019, the Vela Diagnostics NGS platform achieved Food and Drug Administration (FDA) approval for HIV genotyping and resistance testing for in vitro diagnostic purposes [11]. This Ion Torrent technology-based Sentosa^®^ platform allows for the sequencing of HIVDR-relevant genes with sensitivity for minor variant detection at 10% when the viral load (VL) is ≥5000 copies/mL or 20% when VL is lower [9]. Several independent studies have assessed the performance of the Vela Diagnostics NGS platform in research contexts, often with a limited number of selected patients or only with reference materials [12,13].

In this study, we present the results of a comprehensive validation of the Vela Diagnostics NGS system in conjunction with the Sentosa SQ HIV1 genotyping assay, focusing on technical parameters such as the success rate, accuracy, repeatability, and reproducibility of results. The analytical assessment of the Vela NGS platform was conducted in a real-world clinical setting using 35 patient samples and 5 reference samples recommended by College of American Pathologists (CAP). Further, the clinical utility of the assay was underscored by comparative analysis with the current gold standard orthogonal methods—Abbott ViroSeq and Integrase. Our findings demonstrate that the Vela Diagnostics system provides essential insights into the diversity of HIVDR variants, showcasing the transformative potential of NGS methodologies in optimizing clinical management and advancing the collective objective of curtailing the HIV epidemic.

## 2. Materials and Methods

### 2.1. Sample Collection and Processing

The study assessment was guided by the joint consensus recommendation for the validation of NGS assays by the Association of Molecular Pathologists (AMP) and CAP. This retrospective study included 35 unique patient samples with known DRMs on follow-up at the Augusta University Hospital under IRB#611298, approved by the Augusta University Institutional Review Board. All patient identifiers were removed, and all data were anonymized before processing the samples. The blood samples were collected in EDTA tubes (BD Vacutainer^®^, Milan, Italy); plasma fractions were separated with a Cobas p630 pre-analytical instrument (Roche Diagnostics S.p.A., Monza, Italy) and stored at −80 °C until testing. Five CAP-PT (proficiency testing) samples having known DRMs were included as reference samples to evaluate the precision performance of the Sentosa^®^ SQ HIV Genotyping Assay. All these unique 35 samples (run in replicates) were previously analyzed on the Sanger sequencing-based Abbott ViroSeq™ IVD platform for clinical diagnosis. The data were retrieved retrospectively from the patients’ results repository. The samples with a sufficient volume of 750 µL of plasma for testing by NGS and around 500 µL for Sanger sequencing were included in the study. This volume contained sufficient amplifiable RNA for conventional reverse transcription polymerase chain reaction (RT-PCR) and SS in the clinical laboratory.

### 2.2. Dideoxynucleoside Sanger Sequencing

HIV RNA was extracted from plasma specimens utilizing the HIV ViroSeq module, including the provided positive and negative controls inherent in the kit (Celera Diagnostics, Alameda, CA, USA). The genomic segments of interest, encompassing the entirety of the protease gene (codons 1 to 99) and a substantial portion of the RT gene (codons 1 to 335), were selectively amplified to yield a 1.8 kb amplicon. This amplification process was facilitated using the ViroSeq™ HIV-1 genotyping kit (Celera Diagnostics, Alameda, CA, USA) as per the manufacturer’s specifications. The resulting amplicon served as the template for subsequent sequencing, employing seven primers, comprising four forward and three reverse primers. This orchestrated primer design amplified distinct regions of the viral genome in a single step, generating an approximately 1.3 kb consensus sequence. A gel-based, semi-quantitative method was used to determine the quality and concentration of the PCR products by comparing the band intensities to known quantities of standard markers. Notably, 6 and 3 µL of the PCR product were utilized for quantification, with band intensity and PCR product dilution calibrated to ensure accuracy.

After quantification, the PCR product was diluted and subjected to cycle sequencing, employing dideoxy nucleotide triphosphates (dNTPs). The dideoxy products were purified and sequenced on the ABI 3130 genetic analyzer (Applied Biosystems, Carlsbad, CA, USA). The generated consensus sequence was then aligned against a well-established reference sequence, Human Immunodeficiency Virus Type 1 (HXB2), to discern any mutations present within the sample. To streamline this process, the ViroSeq^®^ HIV-1 Genotyping System Software was employed, employing a proprietary algorithm developed by Abbott Molecular Inc. The sophisticated software analyzed the identified mutations, generating a comprehensive drug resistance report for each sample.

The integrase region was reverse-transcribed and amplified following an Integrase sequencing module kit (Celera Diagnostics, Alameda, CA, USA). The entire integrase gene was amplified to generate a 1.1 kb amplicon. The amplicon was used as a sequencing template for four primers (two forward and two reverse primers) that generated an approximately 0.9 kb consensus sequence. The ViroSeq™ Integrase Software (v1.0.1) then assembled, edited, and identified mutations within this 0.9 kb sequence. The software compares the consensus sequence with HXB2 to determine mutations present in the sample and generates a drug report.

### 2.3. NGS and Data Analysis

**Figure 1:**
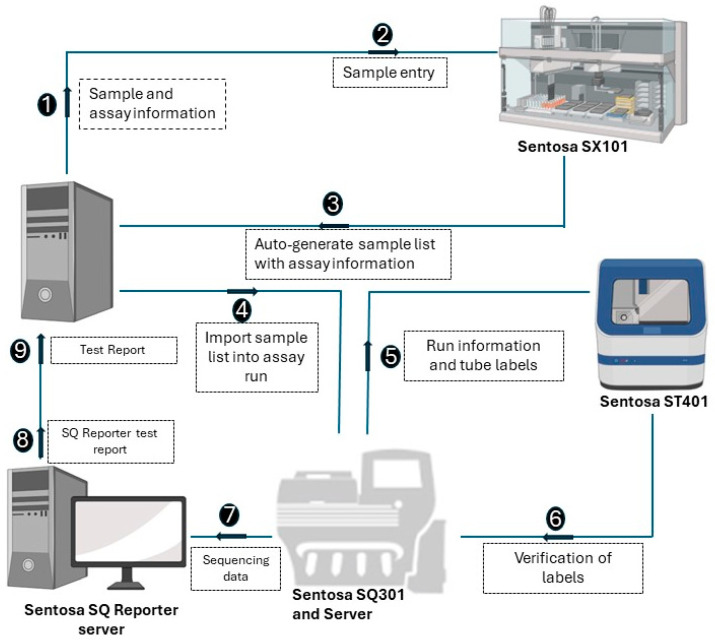
Schematic representation of the steps involved in the workflow of Vela NGS sample preparation, sequencing, and reporting.

The NGS assay simultaneously processes 15 samples, with a positive control in each run. A detailed stepwise procedure is shown in Figure 1. Briefly, RNA extraction from the plasma samples was performed using the Sentosa^®^ SX101 instrument (Vela Diagnostics, Hamburg, Germany), followed by an off-board RT-PCR step using the Veriti^®^ Dx 96-Well Thermal Cycler (Thermo Fisher Scientific, Waltham, MA, USA). Subsequently, the fully automated Sentosa^®^ SX101 instrument constructed a pooled library, which underwent a template preparation on the Sentosa^®^ ST401 system (Vela Diagnostics, Hamburg, Germany). Following an enrichment procedure performed with the Sentosa^®^ ST401e instrument (Vela Diagnostics, Hamburg, Germany), sequencing occurred on the Sentosa^®^ SQ301 (Vela Diagnostics, Hamburg, Germany), based upon the Ion Torrent™ system (Thermo Fisher Scientific, Waltham, MA, USA) for ultra-deep sequencing. An extraction control (EC) was used to monitor RNA extraction and the library preparation steps, while a system control (SC) was analyzed as a sample and provided a check for the entire analysis run. The quality of the SC assembly, i.e., its run throughput (40,000,000 bp), median coverage (200), completeness (95%) and error rate (<1%), is essential to validate the sample results. The integrated workflow includes the automated Sentosa NGS platform (robotic systems and instruments for sequencing) and the Sentosa SQ HIV genotyping assay comprising all the kits for the sequencing of the HIV1 genotyping panel targets protease gene (Codon 1–99), RT gene (Codon 1–376), and Integrase gene (Codon 1–288). HXB2 was used as a reference sequence. The raw signal data, approximately 230 GB per sequencing run, were generated by the sequencer and transferred to the SQ Suite server during the sequencing process. The raw sequencing data were then processed by the SQ Suite server to produce the demultiplexed sequencing data and intermediate files, around 20 GB per run.

### 2.4. Post NGS Drug Susceptibility Reporting Workflow

The Sentosa^®^ SQ Suite and SQ Reporter software packages (v2.5.0005, Vela Diagnostics, Hamburg, Germany) were used for primary and secondary data analysis. The entire workflow generated FASTA files with consensus sequences relevant to the INT region (codons 1–288) and the PRO region (codons 1–99), concatenated with the RT region (codons 1–386). Nucleotide variants > 5% were reported using the International Union of Pure and Applied Chemistry–International Union of Biochemistry and Molecular Biology (IUPAC-IUBMB) system. Beyond raw FASTA files, the assessed subtype based on PRO and RT sequences was provided within a drug susceptibility report. The latter was automatically generated using Sentosa^®^ SQ Reporter software, exploiting the Stanford HIVdb software (v9.0) and Stanford HIVdb 8.2 HIV algorithms. The demultiplexed data from the SQ Suite server were forwarded to the SQ Reporter software for secondary analysis. The SQ Reporter data, totaling about 10 GB per run, are the output of the SQ Reporter analysis pipeline and include sequencing data copies, mapping results, intermediate outputs, and final outputs such as PDF reports, Contigs, and VCF files. Finally, the results dataset, approximately 30 MB per run, comprises the conclusive reportable outputs downloadable via the SQ Reporter interface, encompassing PDF reports, Contigs, VCF files, and various textual formats.

### 2.5. Statistical Analysis

The data obtained were saved in Microsoft Excel and were analyzed using GraphPad Prism 8 (version 8.0.2, San Diego, CA, USA). A Bland–Altman analysis was performed to measure the agreement between the Vela NGS and Abbott ViroSeq systems by plotting the difference in the number of samples in which drug resistance was detected using each assay versus the average of the total number of samples in which mutations were detected using both the assays.

## 3. Results

### 3.1. Patient Characteristics

The general characteristics of the 35 infected patients were as follows: 27 men and 8 women (sex ratio: 0.3), aged from 7 to 64 years (median: 42 years). The HIV subtype found in the samples was mainly B, with a median HIV-1 viral load of 3000 cp/mL and a range of 199,728 cp/mL. In the analysis, we investigated a total of 28 NRTI, 25 NNRTI, 25 PI, and 10 integrase gene unique DRM variants across the 40 (35 test samples and 5 CAP-PT) samples in multiple runs.

### 3.2. Performance Metrics of Vela NGS Sequencing

**Figure 2:**
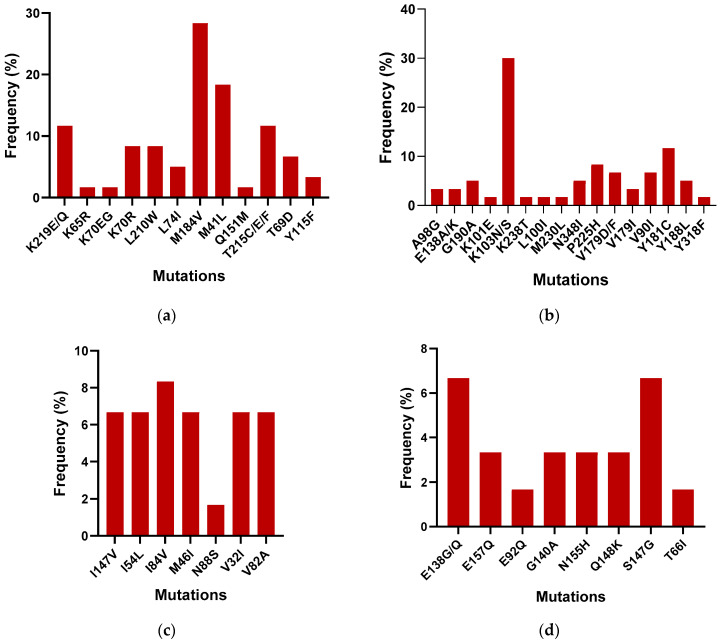
Frequency of the occurrence of DRMs in the (**a**) NRTI, (**b**) NNRTI, (**c**) PI, and (**d**) IN regions among the 40 samples.

To compare the outcomes of the Sentosa^®^ SQ HIV Genotyping Assay with the gold standard Abbott ViroSeq, 40 samples were tested with both the technologies. All samples passed quality controls on the Sentosa^®^ NGS platform. The Sentosa^®^ SQ HIV Genotyping Assay identified the presence of several drug resistance mutations (DRMs) in the reverse transcriptase, protease, and integrase regions. Figure 2 provides a detailed breakdown of the most prevalent DRMs identified by the Sentosa^®^ assay. In the NRTI region, the M184V (30%) mutation was the most frequently observed, followed by the M41L (18.33%) mutation, within the tested samples, leading to reduced susceptibility to 3TC, FTC, AZT, TDF, and D4T. In the NNRTI region, the K103N mutation was most common (28%), followed by Y181C (11.67%), resulting in resistance to the NPV, EFV, ETR, and RPV drugs. However, all mutations exhibited similar frequencies in the protease region except for L10FI and L76V, which confer resistance to ATV, DRV, FPV, IDV, LPV, NFV, and TPV. In the integrase region, the highest frequency of occurrence was associated with the S147G mutation (6.67%), followed by E138G (5%). S147G is known to cause resistance to the EVG drug, while E148G reduces the effectiveness of the ETR, RPV, NPV, and EFV drugs.

### 3.3. Comparison of Vela NGS System and Sanger Sequencing

Complete concordance (100%) was observed between the two sequencing methods for the detection of these 43 DRMs (Table 1). Specifically, 7 DRMs were identified in the PI gene region, 16 in NNRTI, 12 in NRTI, and 8 in the integrase gene region. The proportion of DRMs varied by gene region, with 16.6% of PI variants, 35.7% of NNRTI, 28.57% of NRTI, and 19.04% of integrase variants. Notably, certain mutations were exclusively detected by Vela NGS and not by Abbott. Within the NRTI region, E44D and K70EG were identified, within the NNRTI region, H22Y1, N348I, and V179I were found, and within the integrase region, E92Q was observed solely through NGS and not detected by Abbott.

**Figure 3:**
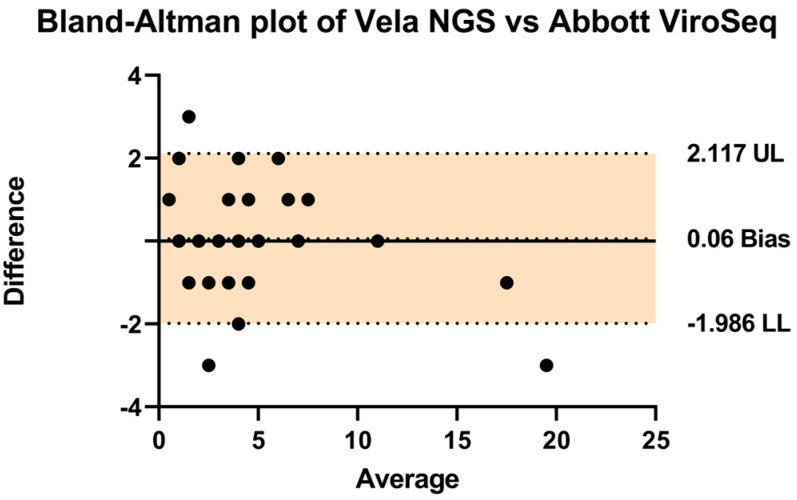
A Bland–Altman plot was used to assess the agreement (how closely the measurements obtained from the two different methods or systems align with each other) between the Vela NGS and Abbott ViroSeq systems by plotting the differences between them against their average. Individual data points, depicted as black dots, represent differences in values obtained from both techniques for identical mutations. The central thick line represents the zero bias, while the dotted lines above and below denote the resulting bias (depicting that one method consistently measures higher or lower values compared to the other method). The clustering of these dots around the zero-difference line indicates an overall statistically significant agreement between the systems. The upper (UL) and lower limits (LL) of agreement are shown by a colored area representing the range within which 95% of the differences between measurements from the two systems fall.

In order to assess the comparative performance of the two systems, a Bland–Altman analysis was conducted as presented in Table 1 (Figure 3). The analysis revealed a negligible bias of 0.06, with upper and lower limits of agreement at 2.11 and −1.986, respectively. These findings indicate a high level of agreement between the Vela NGS platform and the Abbott ViroSeq system in detecting drug resistance mutations.

### 3.4. Precision Analysis of Vela NGS System

Intra-assay evaluations were conducted through repeated runs within the same assay to assess reproducibility, and inter-assay evaluations between different runs to evaluate repeatability, using the Sentosa^®^ SQ301 sequencing instrument.

#### 3.4.1. Determination of Limit of Detection (LOD) and Intra-Assay Reproducibility

The plasma of a patient sample (viral load 18,860 cp/mL) was diluted with 0.9% NaCl to six concentrations (2000 cp/mL, 1000 cp/mL, 750 cp/mL, 500 cp/mL, 250 cp/mL, and 100 cp/mL) which were tested in a run to evaluate LOD and intra-assay repeatability performance (Table 2). In the intra-run analysis, sample 17_5913 was used. Since the Sentosa system requires unique sample codes for adding samples to the run sheet, this sample was assigned different sample IDs for the analysis, as shown in the table. The viral load requirements suggested by the supplier (HIV-1 RNA > 1000 cp/mL) were fulfilled for two out of six samples. However, all samples passed quality control criteria and the Sentosa^®^ NGS platform reported no warning messages. Sequencing analysis of the diluted samples revealed no change in assay sensitivity with sample dilution. It is worth noting that all samples provided overlapping results, with no mismatches in variant detection. The result suggested that drug resistance mutation was reliably detected with a sample input concentration 10-fold lower than that recommended by Vela Dx (1000 copies/mL). A total of nine variants were detected; six were DRMs observed in RT regions.

#### 3.4.2. Inter-Assay Accuracy

To assess the Sentosa^®^ SQ HIV Genotyping inter-assay repeatability, four clinical samples were tested in triplicate (Table 3). In the inter-run analysis, samples 5913, 6049, 6050, and 7072 were used. As explained above, these samples were assigned different names for the analysis, as shown in the table. The patients had a viral load range of 2000–10,000 cp/mL. Overall, 11 DRMs were identified (7 NNRTI and 5 NRTI in the RT region) and 12 minor variants were found, mostly in the protease region. More significantly, no mismatches were detected for all the patients. A complete concordance in inter-assay variant frequencies was observed for each region.

Also, the overall accuracy of the assay was determined by calculating the percentage positive agreement (PPA), negative percentage agreement (NPA), positive predictive value (PPV), and negative predictive value (NPV) for each variant type (Table 4).

## 4. Discussion

In the present study, we conducted a comprehensive validation of the analytical capabilities of the Vela NGS platform, designed for automated HIV genotyping from plasma samples, encompassing the identification and quantification of resistance mutations. We compared the performance of the NGS system against the prevailing gold standard method, the Abbott ViroSeq Sanger sequencing, coupled with the Integrase software. Our evaluation included the technical proficiency of the NGS assay and the potential ramifications of adopting the Sanger sequencing approach on clinical patient management. The technical appraisal revealed the robustness of the Vela HIV-1 genotyping assay, showcasing performance parity with the established Sanger sequencing method in detecting various variants of the viral population. Furthermore, the NGS methodology exhibited an exceptional capability in uncovering additional variants compared to Sanger sequencing. These observations resonate with previous studies, notably Weber et al., whose investigations similarly highlighted the concordance between Sanger and Vela NGS sequencing in detecting DRMs, albeit with the NGS method detecting a higher proportion thereof [14].

The Vela NGS system demonstrated robust detection capabilities, particularly in identifying the majority variants within subtype B at a 100–2000 copies/mL viral load range. In contrast, the Abbott Viroseq HIV-1 Integrase Genotyping kit fails to provide reliable results when the viral load is below 500 copies/mL [15], highlighting a limitation in its detection capability. For instance, while the Genotyping System typically requires a viral load surpassing 2000 copies/mL for effective mutation detection [16], here, we show that the Vela NGS system successfully identified mutations in samples with viral loads as low as 100 copies/mL. By surpassing the limitations of conventional methods, we demonstrate the reliability of the Vela NGS system even in cases when HIV-1 viremia falls below the threshold specified by the manufacturer’s package insert (1000 copies/mL). This underscores its utility in clinical settings for accurate and sensitive HIV-1 mutation detection, facilitating more effective patient management and treatment strategies. The mutations detected at lower levels can help in early detection and thus initiation of therapy for better patient outcomes.

The Vela NGS system also exhibits higher sensitivity in detecting drug resistance mutations (DRMs) compared to Abbott, even when tested in duplicate and triplicate. As shown in Table 1, Vela NGS revealed specific mutations that were not identified by the Abbott assay. Notable mutations within the NRTI region included E44D and K70EG. In the NNRTI region, H22Y1, N348I, and V179I were observed. Additionally, within the integrase region, E92Q was solely identified through NGS, eluding detection by Abbott. The E44D/A are accessory mutations that generally occur with type I TAM (Thymidine analog mutations). These mutations occur in about 1% of viruses from untreated patients and in a significantly higher proportion of viruses from patients receiving NRTI [17]. In the Neubert et al. (2017) study, a patient diagnosed at age 13 harbored a virus carrying the NRTI mutation K70EG along with M184V and the NNRTI mutation K103N [18]. In the NNRTI region, H22Y1 was found to be rare as there was a paucity of data in the literature; however, N348I is a well-established amino acid mutation in connection with the subdomain of HIV1 RT which confers multiclass resistance to NRTI and NNRTI [19]. In the case of V179I, a study found it to be significantly higher in subtype C patients treated with either efavirenz or nevirapine [20]. However, in our study, it was higher in subtype B patients. N155H emerged as a major resistance pathway to first-generation integrase strand transfer inhibitors (INSTIs) within the integrase region, often accompanied by secondary mutations. Anstett et al. demonstrated that while certain mutations like T97A, E157Q, or G163R improved affinity, compensatory mutations such as L74M or E92Q negatively affected enzyme efficiency and viral infectivity in an N155H-R263K background under continued second-generation INSTI pressure [21].

Also, the Bland–Altman analysis revealed a minimal bias of 0.06, with narrow limits of agreement (2.11 and −1.986), indicating a high consistency between Vela NGS and Abbott ViroSeq in detecting drug resistance mutations. This suggests that Vela NGS can serve as a reliable alternative to Abbott ViroSeq. Furthermore, ViroSeq employs distinct kits and software algorithms for HIV and Integrase analysis, necessitating additional procedural steps and potentially elevating error rates. In contrast, the Vela NGS system offers a streamlined workflow encompassing comprehensive analysis within a singular platform. Leveraging automated RNA extraction, precise PCR reagent distribution, seamless library preparation, and advanced bioinformatics analysis, Vela’s solution markedly reduces operational time and minimizes the likelihood of human error. Furthermore, in contrast to other studies comprising Vela NGS system validation [11,12,13], our study successfully obtained genotyping results from all the samples (40/40) with a 100% success rate. Most importantly, we utilized CAP material as the reference material, which is crucial in clinical settings for effectively validating an in vitro diagnostic (IVD) test.

Our analysis reveals specific mutations exclusively detected by Vela NGS, notably within the NRTI, NNRTI, and integrase regions, highlighting its efficacy in identifying diverse mutations critical for treatment selection and patient management. These findings underscore the clinical utility of the Vela NGS system in advancing HIV care, offering enhanced detection sensitivity and a streamlined workflow for improved patient outcomes.

## 5. Limitations and Suggestions

Despite the rigorous methodology employed in this study, there are several limitations which need to be acknowledged.

### 5.1. Sample Size Considerations

Despite the rigorous methodology employed in this study, the sample size used for validation could have been expanded. Future studies should aim to validate findings on a larger, more diverse cohort to enhance statistical power and generalizability.

### 5.2. Focus on Subtype B

This study primarily discusses drug resistance in HIV subtype B, limiting insights into other HIV subtypes. To broaden applicability, research should encompass additional HIV subtypes beyond subtype B, such as A, C, and recombinant forms.

### 5.3. Generalizability Issues

The findings may be limited by specific cohort characteristics, such as demographics and geographic location. Efforts to replicate findings in diverse populations will validate the robustness of the NGS system across varied contexts.

## 6. Conclusions

The study unequivocally validates the efficacy of the Vela Diagnostics Ion Torrent-based NGS platform for HIV-1 genotyping. Demonstrating comparable performance to the established Sanger sequencing method, the Vela system offers an additional advantage by detecting additional DRMs and has a streamlined workflow, thereby enhancing HIV-1 patient management strategies. Furthermore, the system’s integrated data analysis represents significant advancements, rendering the technology more accessible to laboratories lacking in-house expertise for laborious workflows or intricate bioinformatics analyses typically associated with NGS methodologies. This accessibility democratizes advanced NGS genotyping techniques, promising to broaden their application and impact across diverse clinical settings.

## Figures and Tables

**Table 1:** Summary of all drug resistance mutations detected in patient samples by Vela NGS and Abbott ViroSeq.

Mutation	No. of Patient Samples Positive for the Mutation		Gene Regions
	Vela NGS	Abbott ViroSeq	
E138G/Q	4	4	IN
E157Q	2	2	IN
E92Q	1	0	IN
G140A	2	3	IN
K156N	1	1	IN
L74M	1	1	IN
N155H	2	2	IN
Q148K	2	3	IN
S147G	4	4	IN
T66I	1	1	IN
T97A	1	1	IN
A98G	2	2	NNRTI
E138A/K	2	2	NNRTI
G190A	3	5	NNRTI
H221Y	2	0	NNRTI
K101E	1	1	NNRTI
K103N/S	18	21	NNRTI
K238T	1	1	NNRTI
L100I	1	2	NNRTI
M230L	1	1	NNRTI
N348I	3	0	NNRTI
P225H	5	3	NNRTI
V106I	3	4	NNRTI
V108I	1	1	NNRTI
V179D/F	4	4	NNRTI
V179I	2	0	NNRTI
V90I	4	4	NNRTI
Y181C	7	5	NNRTI
Y188L	3	3	NNRTI
Y318F	1	1	NNRTI
A62V	1	1	NRTI
D67N	8	7	NRTI
E44D	2	0	NRTI
F116Y	1	1	NRTI
F77L	1	1	NRTI
K219E/Q	7	6	NRTI
K65R	1	1	NRTI
K70EG	1	0	NRTI
K70R	5	4	NRTI
L210S	1	0	NRTI
L210W	5	5	NRTI
L74I	3	4	NRTI
M184I	4	4	NRTI
M184V	17	18	NRTI
M41L	11	11	NRTI
Q151M	1	1	NRTI
T215C/E/F	7	7	NRTI
T69D	4	5	NRTI
T69N	3	3	NRTI
T69S	2	3	NRTI
V75I	1	1	NRTI
Y115F	2	2	NRTI
I147V	4	3	PI
I54L	4	4	PI
I84V	5	5	PI
L10FI	1	4	PI
L90M	1	1	PI
M46I	4	4	PI
N88S	1	1	PI
V32I	4	4	PI
V82A	4	4	PI

**Table 2:** Evaluation of limit of detection (LOD) and intra-assay repeatability performance for various viral load concentrations.

Run ID	Sample ID	Viral Load (cp/mL)	NRTI	NNRTI	PI	INT
1_ENEWL	18-1628	2000	K65R, Y115F	K101E, E138Q, V179F, Y181C	Other: M36I, L63P	Other: K156N
1_ENEWL	18-1629	1000	K65R, Y115F	K101E, E138Q, V179F, Y181C	Other: M36I, L63P	K156N
1_ENEWL	18-1630	750	K65R, Y115F	K101E, E138Q, V179F, Y181C	Other: M36I, L63P	K156N
1_ENEWL	18-1631	500	K65R, Y115F	K101E, E138Q, V179F, Y181C	Other: M36I, L63P	K156N
1_ENEWL	18-1632	250	K65R, Y115F	K101E, E138Q, V179F, Y181C	Other: M36I, L63P	K156N
1_ENEWL	18-1633	100	K65R, Y115F	K101E, E138Q, V179F, Y181C	Other: M36I, L63P	K156N

**Table 3:** Inter-assay accuracy for various viral load concentrations in replicates.

Run ID	Sample ID	Viral Load	NRTI	NNRTI	PI	INT
1_ENEWL	18-1628 (17-5913)	2000	K65R, Y115F	K101E, E138Q, V179F, Y181C	Others: M36I, L63P, I62V	Other: K156N
2_LKED2	18-4707 (17-5913)	2000	K65R, Y115F	K101E, E138Q, V179F, Y181C	Others: M36I, L63P, I62V	Other: K156N
3_QPWOE	18-4757 (17-5913)	2000	K65R, Y115F	K101E, E138Q, V179F, Y181C	Others: M36I, L63P	Other: K156N
1_ENEWL	18-1635 (17-6049)	4000	M184V	Other: V901I, V118I	Other: V77I	E92Q Other: K156N
2_LKED2	18-4708 (17-6049)	8277	M184V	Other: V901I, V118I	Other: V77I	K156N
3_QPWOE	18-4758 (17-6049)	8277	M184V	Other: V901I, V118I	Other: V77I	K156N
1_ENEWL	18-1636 (17-6050)	10,000	-	V179D, G190A, Y318F Other: V90I, V106I	Other: M36I, L63P	Other: T206S
2_LKED2	18-4709 (17-6050)	10,000	-	V179D, G190A, Y318F Other: V90I, V106I	Other: M36I, L63P	Other: G193E, T206S
3_QPWOE	18-4759 (17-6050)	10,000	-	V179D, G190A, Y318F Other: V90I, V106I	Other: M36I, L63P	Other: T206S
1_ENEWL	18-1639 (17-7072)	10,000	M41L, T215E	-	Others: M36I, L63P, I64LM, I93L	-
2_LKED2	18-4710 (17-7072)	10,000	M41L, T215E	-	Others: M36I, L63P, I64LM, I93L	-
3_QPWOE	18-4760 (17-7072)	10,000	M41L, T215E	-	Others: M36I, L63P, I64LM, I93L	-

**Table 4:** Overall accuracy (sensitivity and specificity) determination of the assay by calculating positive and negative agreement and predictive values (*n* = 35 samples).

	PPA = TP/(TP + FN) (Sensitivity)	NPA = TN/(TN + FP) (Specificity)	PPV = TP/(TP + FP)	NPV = TN/(TN + FN)
NRTI DR Mutation	100%	100%	100%	100%
NNRTI DR Mutation	100%	100%	100%	100%
PI DR Mutation	100%	100%	100%	100%
Integrase	100%	100%	100%	100%

TP = True Positive (positive outcome when the mutation is indeed present); TN = True Negative (negative outcome when the mutation is indeed absent); FP = False Positive (incorrectly indicates the presence of a mutation when it is absent); FN = False Negative (incorrectly indicates the absence of a mutation when it is present); PPV = Positive Predictive Value (the likelihood that a positive mutation detection result accurately indicates the presence of that mutation); NPV = Negative Predictive Value (the likelihood that a negative mutation detection result accurately indicates the absence of that mutation).

## Data Availability

The original contributions presented in the study are included in the article, further inquiries can be directed to the corresponding author.
